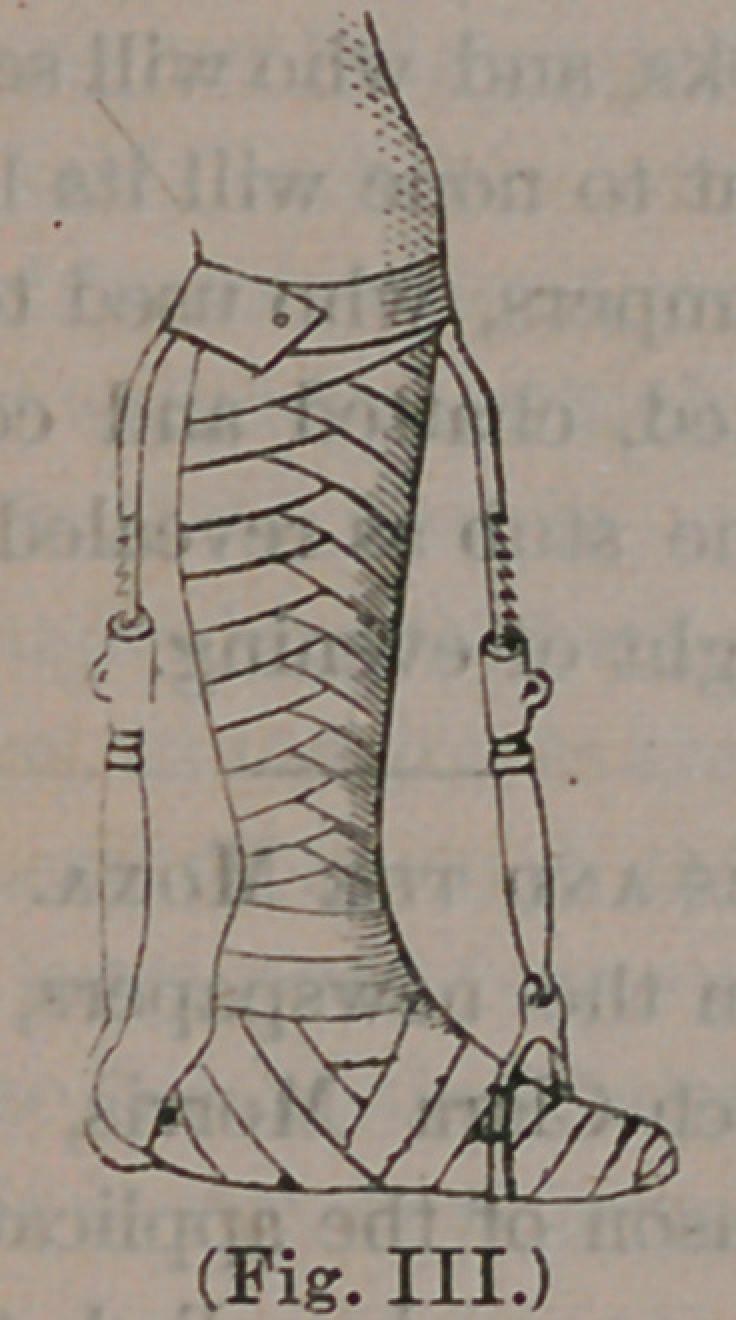# Ankle Joint

**Published:** 1875-10

**Authors:** 


### ANKLE JOINT.

It has become quite common to see children and young men hobble about on crutches, by reason of swollen and painful joints. The knee and ankle joints are subject to injuries and diseases, which, if not properly cared for, result most disastrously, often crippling the subject for life. The ankle, when attacked with tubercular or scrofulous inflammation, causes great swelling, with a soft or puffy feeling, from the accummulation of the synovial fluid ("joint water") which distends the parts. As the disease progresses, the ends of the long bones of the leg become involved in the disease, softening, and finally ulcerating, when an abscess forms and opens upon the ankle, as here represented:

(Fig. I.)

This disease is usually easily arrested in the beginning, and it is for this reason that we call the attention of parents to it, so that they can avail themselves early of the means of cure.

Strips of adhesive plaster, about an inch wide, and reaching from just above the ankle to the knee, are placed all about the leg, as illustrated in Fig. 1. A roller bandage is then applied over these strips to hold them in position, to within an inch or two of the top. Then an instrument like the following is applied:

(Fig. II.)

consisting of a stout steel plate, cut to the exact size of the sole of the foot. At the heel is a joint to which is fastened a steel rod, which terminates near the knee in an adjustable steel band. Immediately over the instep is a stirrup-like attachment, with hinge joint, to which is attached another rod which communicates with the steel band, in front. Both these rods pass up behind and in front of the leg, and are divided in their centres, and arranged with a ratchet, so that they can be lengthened or shortened. The rods are now shortened, and the sole of the foot placed upon the steel sole, and confined there with adhesive plaster and a roller bandage. Then, the band at the top is buckled snugly about the leg, the plasters that were left protruding above, brought over the top of the rim, and confined with the roller that passes up the leg; so that when the operation is completed, it will appear as follows:

(Fig. III.)

The instrument being in position, a key is applied to the ratchet work, by means of which the rods are lengthened, so extending the limb, or drawing the foot away from the ankle, relieving the pressure of the diseased bones against each other. By this arrangement, all pain and irritation is immediately quieted, and the little sufferer will soon fall asleep by reason of the comfort afforded. The apparatus is worn in the incipient stages of the disease, continually, day and night, until all trace of inflammation, tenderness, or swelling, has subsided, the patient going the while on crutches. No other treatment, save perhaps the administration of tonics of iron or quinia, under the direction of the family physician, is needed, until the cure is complete. Where the disease has progressed to suppuration, other local treatment beside the apparatus may be necessary, which must be prescribed by some competent surgeon.

## Figures and Tables

**(Fig. I.) f1:**
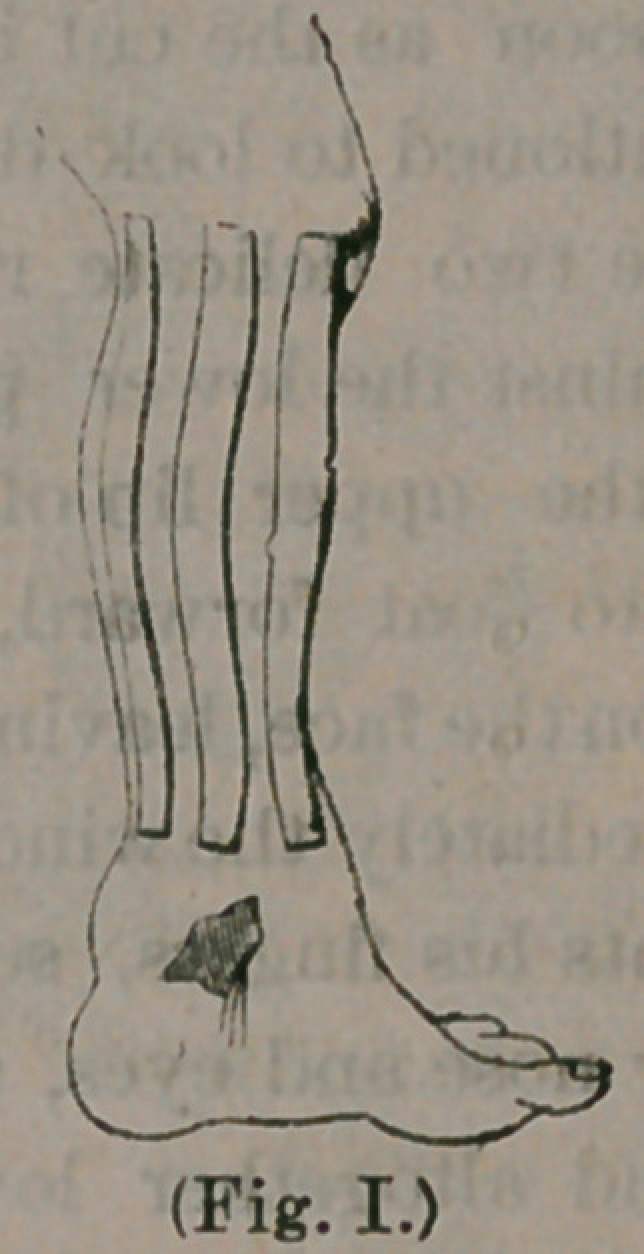


**(Fig. II.) f2:**
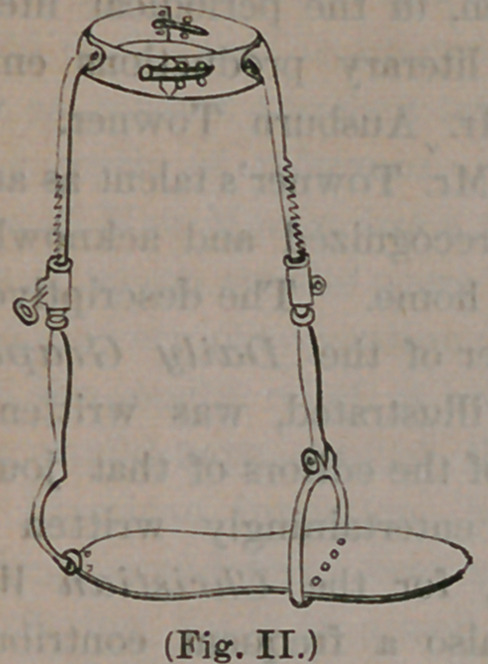


**(Fig. III.) f3:**